# White lung with milky effusion

**DOI:** 10.1002/ccr3.3207

**Published:** 2020-08-09

**Authors:** Tamer Mohamed Zaalouk, Zouheir Ibrahim Bitar, Ossama Sajeh Maadarani, ALAsmar Mohammed El‐shably

**Affiliations:** ^1^ Critical Care Unit Ahmadi Hospital Kuwait Oil Company Ahmadi Kuwait

**Keywords:** acute medicine, critical care medicine, respiratory medicine

## Abstract

Thoracic duct injury is a rare complication of dorsal spine operations. Ultrasound chest plays an important tool for rapid diagnosis of acute dyspnea, drainage of massive effusion, and daily follow‐up. Conservative treatment of postoperative chylous with measures to decrease chylous formation can lead to a resolution of chylothorax.

## Graphical abstract

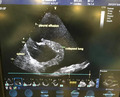

## 1 CASE REPORT

A 71‐year‐old‐woman admitted with acute low back pain of 8 weeks that became severe 3 days before admission. Pain is limited to left lower costal margin with no midline or paravertebral pain. There was tenderness at thoracolumbar junction and over lower ribs and left paraspinal muscles. Reflexes and motor power were normal in four limbs. No cranial nerves affection.

MRI (magnetic resonance image) spine showed D8‐9 (Dorsal) spondylodiscitis with soft tissue collection. Patient underwent surgical debridement and fixation form D6‐D7 to D10‐D11 with fusion D8‐9, through direct dorsal approach. Fixation of transpedicular screws diameter 6.5 length 35mm in dorsal and 60mm diameter, length 40 mm in lumbar, Medtronic. decompression then debridement of D8‐9‐disk level bilaterally.

**Figure 1:**
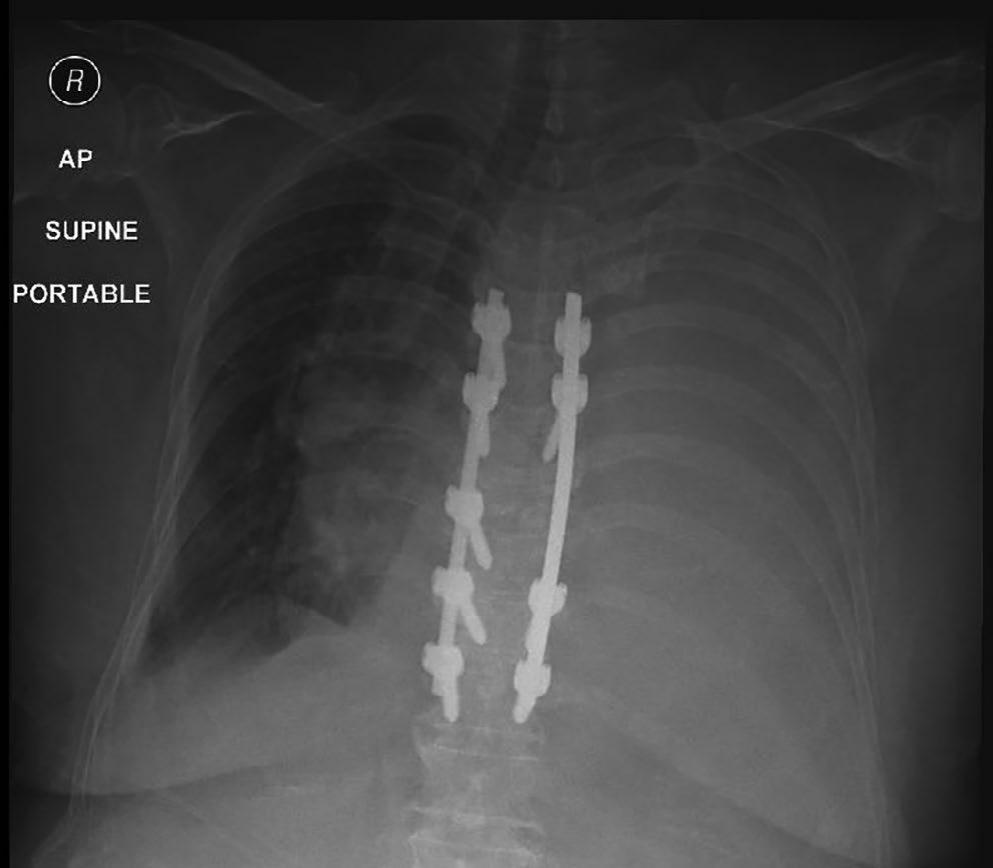
CXR (Chest x‐ray) shows opacification of left lung and dorsal spine fixation

**Figure 2:**
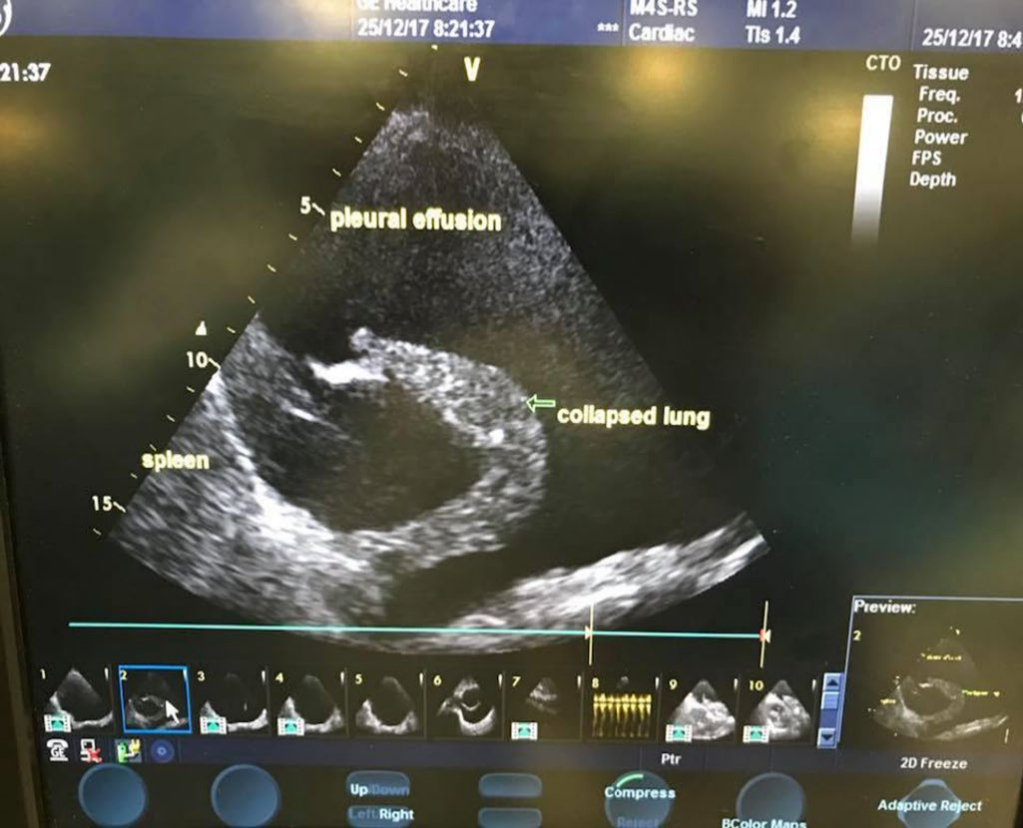
ultrasound chest shows Left pleural effusion. This scan shows substantial effusion with multiple echoes, mobile, and whirling in real‐time (plankton sign). The lower lobe is consolidated

**Figure 3:**
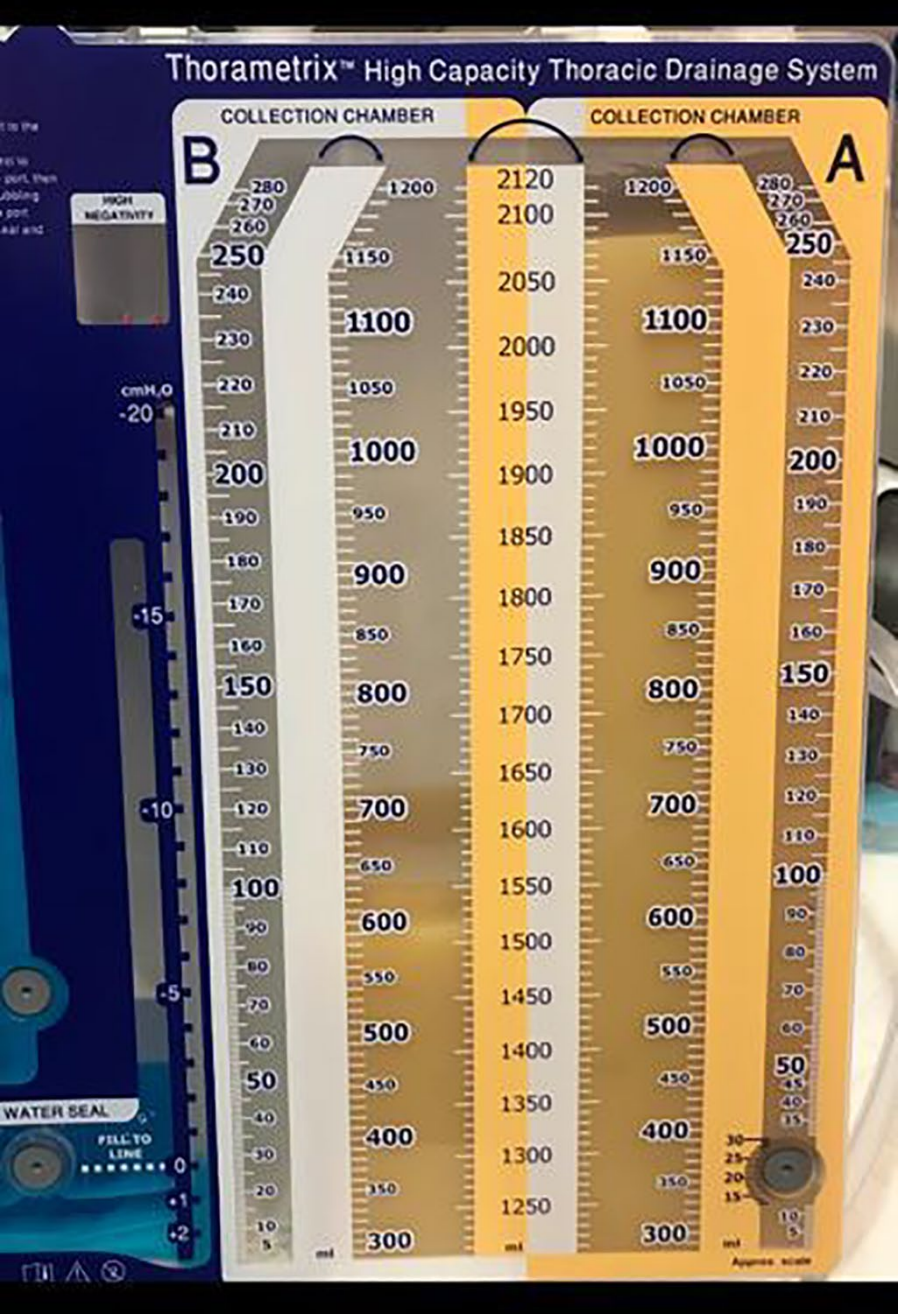
shows chylous effusion

On day 3 postoperatively, patient had progressive dyspnea and CXR (Chest x‐ray) showed complete opacification of the left lung (Figure 1). Urgent chest ultrasound showed massive left pleural effusion with Plankton sign (Figure 2). Chest tube, pigtail size 8 French, was inserted at intercostal space to drain cloudy milky fluid (Figure 3). 2.5 liters of milky fluid was drained after 24 hours. Pleural fluid showed high triglyceride 800 mg/mL (10.10 mmol/L); total protein was 36.8 g/L and total cholesterol 180 md/dl (4.8 mmol/L).

Chylothorax was confirmed. The patient was kept NPO (Nothing per mouth), TPN (Total parenteral nutrition) started, and somatostatin infusion. Left pleural effusion was decreased in subsequent days.

Follow‐up CT (computerized tomography) of the chest showed—Multiple spinal fixation screws in the D6 TO D10 vertebral bodies. Left D6 screw was seen extending via the pedicle into the mediastinum, just medial to the descending aorta and touching the esophagus. Left D8 screw is not visualized. Both D9 and D10 screw on the left side is seen penetrating the anterior vertebral cortex extending into the prevertebral soft tissue. Multiple bony fragments were noted in the left half D8 vertebral body.

The chest tube was removed after 5 days with drainage less than 50 mL/d for 2 days. A follow‐up chest ultrasound showed minimal effusion. The patient was discharged home in a stable condition.

**Table:** 

Laboratory results	
Blood tests results	Pleural fluid analysis
WBCs (White blood count) = 11 000	Total body fluid protein = 36.8 gram/liter
HB (Hemoglobin) = 11 grams	Fluid LDH (Lactate dehydrogenase) = 154 μ/L
Platelets = 230 000	Fluid adenosine deaminase = 1.5 U/L
Urea = 15 miligram/dL	Triglyceride level 10.1 mmol/L
Creatinine = 1 miligram/dL	
Total protein = 7 grams/dL	

## 2 DISCUSSION

Chyle is lymphatic fluid drained from the intestine to the bloodstream through the thoracic duct. Chyle has a milky appearance that disappears during fasting due to the high content of triglycerides in the form of chylomicrons and the opalescent appearance of lymphatic fluid. It also has bacteriostatic activity as it contains lymphocytes (primarily T lymphocytes) as the major cellular component (>70%).^1^ The thoracic duct passes through the mediastinum, where it receives nonchylous lymph from tributaries that drain regions of the pulmonary parenchyma and parietal pleura.^2^ The sum of these sources accounts for a total lymphatic flow through the thoracic duct of 1500‐2400 mL/d.^3^

Chylothorax can be traumatic or nontraumatic with equal incidence.^4^ The etiology of chylothorax likely varies by the patient population managed in the reporting institution. In traumatic chylothorax, disruption of the thoracic duct or its tributaries anywhere along their course can cause chylothorax.^5^ Surgical procedures, such as Esophagectomy,^6^ pulmonary resection with lymph node dissection,^7^ surgery for congenital heart disease including heart‐lung transplantation in the thoracic duct or nearby structures, which account for majority of cases of traumatic chylothorax, can disrupt the thoracic duct or tear lymphatic tributaries.^8^, ^9^ Many reported cases is connected to complicated thoracic or abdominal procedure.^4^, ^10^

The milky‐appearing fluid suggests the presence of chylothorax, but other differential diagnoses should be considered, for example, cholesterol pleural effusion, or an empyema. The WBCs count is elevated but mainly lymphocytes of a polyclonal population of T cells, and this can rule out empyema. Pleural fluid triglyceride content measurement is the initial test to diagnosis a chylothorax. A pleural fluid triglyceride concentration greater than 110 mg/dL (1.24 mmol/L) strongly supports the diagnosis.^11^ Lipoprotein electrophoresis of pleural effusion is reserved for patients with an intermediate pleural fluid triglyceride level between 50 mg/dL and 110 mg/dL.^12^ In our case, the level was high, so there was no need for electrophoresis. The cholesterol level in a chylothorax is generally less than 200 mg/dL (5.18 mmol/L).^12^

Usually, an injury to the thoracic duct at or below the fifth thoracic vertebra results in a right‐sided chylothorax, and injury above the fifth thoracic vertebra would result in a left‐sided chylothorax.^13^ However, an anatomic variation of the normal course, which is not uncommon, could explain the left‐sided chylothorax in our patient.^14^ Bhat et al reported 3 cases of Chylous leakage is an uncommon complication following anterior spinal surgery. It followed thoracic duct, cisterna chyli or retroperitoneal lymphatic vessel injury. All cases were managed nonoperatively.^15^ In the Multicenter retrospective case series, 9591 patients reviewed that underwent cervical spine surgery, only 2 cases (0.02%) had an iatrogenic injury to the thoracic duct.^16^ Both patients underwent a left‐sided anterior cervical discectomy and fusion and managed conservatively.

Depending on the volume of chyle loss, chylothorax can be low‐output chylothoraxes (<1000 chyle drainage per day) or large volume drainage (>1 L per day) of chylous. Large leaks can cause nutritional deficiencies, respiratory dysfunction, dehydration, and immunological dysfunction.^17^ Conservative treatment is recommended initially for post‐traumatic chylothorax. Standard methods include chest tube drainage, dietary fat restriction, and total parenteral nutrition. If these prove ineffective, somatostatin can be given by continuous infusion. Somatostatin is a peptide hormone that acts as a neurohormone as well as a paracrine agent. The exact mechanisms involved in the drying effect of somatostatin on lymphatic leakage are not wholly understood. The effectiveness of somatostatin may be due to its ability to decrease the hepatic venous pressure gradient, to decrease the intestinal absorption of fats, to decrease the triglyceride concentration in the thoracic duct, and to attenuate splanchnic blood flow. Somatostatin was given at a dose of 10 µg/kg/hr Patients draining more than 1 L/d are unlikely to respond to conservative therapy and usually require surgical intervention usually thoracic duct ligation within 5‐7 days.^18^ In our patient, the lymphatic drainage was less than 500 mL of chest tube drainage after initiation of medical treatment and did not require surgical intervention.

## 3 CONCLUSION

Thoracic duct injury is a rare complication of dorsal spine operations. Conservative treatment in postoperative chylous leakage is an option with measures to decrease chylous formation which can lead to a complete resolution of chylothorax.

## CONFLICT OF INTEREST

None declared.

## AUTHOR CONTRIBUTIONS

TZ wrote the article, ZIB and OSM shared in the discussion and with AAME in collecting the data and revision of the manuscript.
